# Supplementary material for: Multilayered SDN security with MAC authentication and GAN-based intrusion detection
Source: PLoS One. 2025 Sep 4;20(9):e0331470. doi: 10.1371/journal.pone.0331470 (PMC12410795; doi:10.1371/journal.pone.0331470)
Supplement: S1 Table — (DOCX) [file pone.0331470.s001.docx]

**S1 Table.** **Input and Selected features for data packet transmission**

| Features | Selected features |
| --- | --- |
| Duration  protocol_type  service  flag  src_bytes  dst_bytes  land  wrong_fragment  urgent  hot  m_failed_logins  logged_in  num_compromised  root_shell  su_attempted  num_root  num_file_creations  num_shells  num_access_files  num_outbound_cmds  is_host_login  is_guest_login  count srv_count  serror_rate  srv_serror_rate  rerror_rate  srv_rerror_rate  same_srv_rate  diff_srv_rate  srv_diff_host_rate  dst_host_count  dst_host_srv_count  dst_host_same_srv_rate  dst_host_diff_srv_rate  dst_host_same_src_port_rate  dst_host_srv_diff_host_rate  dst_host_serror_rate  dst_host_srv_serror_rate  dst_host_rerror_rate  dst_host_srv_rerror_rate | protocol_type  service  flag  src_bytes  dst_bytes  logged_in  count srv_count  serror_rate  srv_serror_rate  rerror_rate  srv_rerror_rate  same_srv_rate  diff_srv_rate  srv_diff_host_rate  dst_host_count  dst_host_srv_count  dst_host_same_srv_rate  dst_host_diff_srv_rate  dst_host_same_src_port_rate  dst_host_srv_diff_host_rate  dst_host_serror_rate  dst_host_srv_serror_rate  dst_host_rerror_rate  dst_host_srv_rerror_rate |
