# Supplementary material for: Multilayered SDN security with MAC authentication and GAN-based intrusion detection
Source: PLoS One. 2025 Sep 4;20(9):e0331470. doi: 10.1371/journal.pone.0331470 (PMC12410795; doi:10.1371/journal.pone.0331470)
Supplement: S1 Fig — (DOCX) [file pone.0331470.s002.docx]

| **S1 Algorithm.** GSOM Algorithm for Categorizing Suspicious Packets |
| --- |
| **Input:** Suspicious packets  **Output:** Classified packets (normal, malicious)  **Initialization phase**  Initialize weight factors for each node  **Calculate** the growth threshold: $\boldsymbol{GT=-D\times In(SF)}$  **Growing Phase**  For each input, find winning node $\boldsymbol{N}_{\boldsymbol{K+1}}$  Adjust the weight of the winning node using Eqn. (4)  **Repeat** this process until the packet growth reaches a minimum value.  **Smoothing Phase**  Separate suspicious packets into normal and malicious packets.  **End** |
