# Supplementary material for: Multilayered SDN security with MAC authentication and GAN-based intrusion detection
Source: PLoS One. 2025 Sep 4;20(9):e0331470. doi: 10.1371/journal.pone.0331470 (PMC12410795; doi:10.1371/journal.pone.0331470)
Supplement: S1 File — GSOM algorithm for categorizing suspicious packets. (DOCX) [file pone.0331470.s003.docx]

**S1 Fig. Flowchart of the sheep flock optimization algorithm**
