# Supplementary material for: Multilayered SDN security with MAC authentication and GAN-based intrusion detection
Source: PLoS One. 2025 Sep 4;20(9):e0331470. doi: 10.1371/journal.pone.0331470 (PMC12410795; doi:10.1371/journal.pone.0331470)
Supplement: Attachment — Submitted filename: Reponse to Reviwer Comments (Plos One).docx [file pone.0331470.s006.docx]

**Rebuttal Letter**

Dated: 08/12/2024

To

Academic Editor
PLOS ONE

Subject: Response to Reviewers' Comments for Manuscript titled: **“An authentication system based on MAC addresses for multilayered SDN intrusion detection using dual discriminator conditional GAN and growing self-organizing maps**” with Manuscript Id **PONE-D-24-24864.**

**New Title: “Multilayered SDN Security with MAC Authentication and GAN-Based Intrusion Detection”**

Dear Dr. Muhammad Anwar,

We thank you and the reviewers for the opportunity to revise and resubmit our manuscript, titled “**An authentication system based on MAC addresses for multilayered SDN intrusion detection using dual discriminator conditional GAN and growing self-organizing maps”** submitted to PLOS ONE. We appreciate the reviewers’ thoughtful and constructive comments, which have helped us improve the quality and clarity of our work. We have provided the detailed responses to each comment and explain the revisions made in the manuscript in the file titled ***Response to the Reviewer Comments*.**

We have addressed each comment in turn, and where necessary, we have highlighted the changes in the revised manuscript. Please find our point-by-point responses below. We have also made minor edits to improve clarity and coherence throughout the manuscript. These include rewording certain sentences and correcting typographical errors. We hope that our responses and the revised manuscript address the reviewers’ concerns satisfactorily. We are confident that the changes have significantly improved the paper and would make you give a decision in favour to our proposal. Thank you for your time and consideration, and we look forward to your feedback.

Sincerely,

Nanavath Kiran Singh Nayak, Dr. Budhaditya Bhattacharyya* (Corresponding Author)
School of Electronics Engineering,

Vellore Institute of Technology, Vellore, India.

[budhaditya@vit.ac.in](mailto:budhaditya@vit.ac.in)

+91-9955658860.

**Response to the Reviewer Comments**

**Reviewer #1: Comments:**

1. The current title, "An authentication system based on MAC addresses for multilayered SDN intrusion detection using dual discriminator conditional GAN and growing self-organizing maps," is quite detailed but could be more concise and specific. Here are some suggestions to make the title more focused and clearer:

a. "MAC Address Authentication for SDN Security: A Dual Discriminator GAN and Self-Organizing Maps Approach".

b. "Intrusion Detection in SDN Using MAC-Based Authentication and Conditional GANs"
c. "Multilayered SDN Security with MAC Authentication and GAN-Based Intrusion Detection"

**Response:** As per the reviewer’s valuable comments, we propose adopting "Multilayered SDN Security with MAC Authentication and GAN-Based Intrusion Detection" as the new title. This suggestion aligns with the study’s emphasis on multilayered SDN security, the use of MAC authentication, and the application of GAN-based intrusion detection techniques while maintaining clarity and conciseness.

2. Although the abstract is clearly organized, there are several unclear passages. Change the words from "Computer networks are susceptible to cyber-security intrusions" for "Computer networks are highly vulnerable to cyber-security intrusions." Additionally, as some readers may not be aware with the term "Four-Q curve authentication," quickly define it for them.

**Response:** Thank you for your insightful feedback. We have revised the abstract as per your suggestions. The phrase "Computer networks are susceptible to cyber-security intrusions" has been replaced with "Computer networks are highly vulnerable to cyber-security intrusions" to better align with the intended emphasis. Additionally, a brief definition of "Four-Q curve authentication" has been included in the abstract to ensure clarity for readers unfamiliar with the term.

3. Further background on SDN and its significance in the modern networking environment has to be included in the introduction. Provide a succinct explanation of the drawbacks of conventional SDN designs as well as the importance of multilayer SDN in improving network security.

**Response:** As per the reviewer’s valuable comments, we have revised the introduction Section 1 (Paragraph 2 and 4) to include additional background on SDN and its importance in the modern networking environment. We have also incorporated a concise explanation of the limitations of conventional SDN designs and highlighted the role of multilayer SDN in enhancing network security.

4. The literature study needs to provide a comparative examination of the many strategies that have been discussed. Talk about each method's benefits and drawbacks in further depth. Furthermore, it is important to include new research conducted beyond 2020 in order to maintain the currency of the literature review.

**Response:** As per the reviewer’s valuable comments, we have included Table 1 with detailed benefits, drawbacks, and added recent studies (References 31–33), and updated the literature review.

5. The methodology part needs to have an elaborate flowchart or diagram that demonstrates the architecture of the suggested system. This will make the procedure easier for readers to see and comprehend the processes needed.

**Response:** As per the reviewer’s valuable comments, we have included the flow chart (Figure 4) at the end of the methodology section to visually demonstrate the architecture of the suggested system.

6. Give a more thorough description of the relevance of the "Four-Q curve authentication" procedure in the suggested system.

**Response:** Thank you for your feedback. We have provided a thorough explanation of the relevance of the "Four-Q curve authentication" procedure of the proposed system in section 3.4.

7. The DC-GAN technique should be explained in greater detail. Provide explanations of the dual discriminator's operation and how it improves intrusion detection accuracy using mathematical formulas and algorithms.

**Response:** As per the reviewer’s valuable comments, we have provided a more detailed explanation of the DC-GAN technique in Sections 3.5.2 and 3.5.3. Specifically, we have elaborated on the operation of the dual discriminator. We have also clarified how the dual discriminator improves intrusion detection accuracy by minimizing adversarial loss and refining the detection process. These additions should provide a clearer understanding of the technique's impact on the proposed system.

8. Give a thorough explanation of the Sheep Flock Optimization Algorithm (SFOA). Explain its function in DC-GAN optimization and the rationale behind selecting this specific method over alternative optimization strategies.

**Response:** Thank you for your valuable comment. we have provided a more thorough explanation of the Sheep Flock Optimization Algorithm (SFOA) in the revised manuscript (Section 3.5.3). We also justify the selection of SFOA over alternative optimization methods by highlighting its advantages. This makes SFOA an ideal choice for the proposed system compared to other conventional optimization techniques.

9. The ramifications of the results should be covered in more detail in the discussion section. Talk about the practical applications of the suggested system and how it could affect SDN security.

**Response:** As per the reviewer’s valuable comments, we have included a more detailed analysis of the ramifications of the results and tables in the performance analysis section 4.3. Specifically, we now explore the practical applications of the proposed system in enhancing SDN security (section 4.3).

10. Discuss any potential drawbacks or restrictions with the suggested system and offer ideas for workarounds or new directions for investigation.

**Response:** Thank you for your feedback. At the end of Section 5, we briefly discuss potential drawbacks, and hybrid approaches as workarounds for future exploration.

11. The main conclusions and contributions of the study should be succinctly summarized in the conclusion. Stress the newness and importance of the suggested system for improving SDN security.

**Response:** Thank you for your feedback. Section 5 now succinctly summarizes our study's contributions, emphasizing the proposed system's novelty and its significant impact on enhancing SDN security against modern cyber threats.

12. Provide targeted recommendations for future research trajectories to improve the suggested system even further and fill up any gaps.

**Response:** Thank you for your feedback. At the end of Section 5, we included targeted recommendations for future research.

**Reviewer #2: Comments**

1. Authors should provide some brief numerical results at the end of the abstract to demonstrate that the proposed approach achieves SOTA performance.

**Response:** Thank you for your suggestion. We have updated the abstract to include brief numerical results demonstrating that the proposed approach achieves state-of-the-art (SOTA) performance. These results highlight key metrics such as accuracy, F1-score, Precision, True Positive Rate (TPR), False Alarm Rate (FAR) and energy saving, showcasing the effectiveness of our system compared to existing methods.

2. The authors should provide the reference when DCGAN was first introduced, rather than waiting until line 247. In addition, reference [33] is commonly known as DDcGAN, so why do the authors refer to it as DCGAN?

**Response:** Thank you for your observation. DC-GAN is our proposed approach, so we did not provide a citation when first introduced. However, reference [33] (Now it is the Reference 36 in the manuscript) describes the architecture of the generator and discriminator, which is why it is cited in Section 3 to provide context.

3. Abbreviations (e.g. MAC, DDoS, MLP, IoT, and others) need to be defined before being mentioned for the first time.

**Response:** Thank you for pointing this out. We have reviewed the manuscript and ensured that all abbreviations, such as MAC (Media Access Control), DDoS (Distributed Denial of Service), MLP (Multi-Layer Perceptron), IoT (Internet of Things), and others, are defined when first mentioned in the text. This ensures clarity and helps readers unfamiliar with these terms better understand the content.

4. Each symbol in Equation 2 should be introduced, and its corresponding value and meaning should be clearly explained.

**Response:** Thank you for your feedback. We have revised the manuscript to ensure that each symbol in Equation 2 is clearly introduced and its corresponding value and meaning are explained in detail.

5. The paper lacks figures, and the authors should add them to paper.

**Response:** Thank you for your suggestion. We have addressed this by including relevant figures (Such as Figure 2 and 4 of the Figures files document) to enhance the manuscript.

6. F1-score is a metric that assesses model performance on a scale from 0 to 1. For instance, better to express the F1-score as 0.975 rather than 97.5%.

**Response:** Thank you for the observation. We have revised the manuscript to express the F1-score consistently on a scale from 0 to 1, aligning with standard conventions. For example, the F1-score is now reported as 0.975 instead of 97.5%, ensuring clarity and adherence to best practices in presenting performance metrics.

7. This paper lacks tables of experimental results.

**Response:** Thank you for the observation. Tables (5, 6, 7, and 8) summarizing the experiments results have been included in section 4.3.

8. Authors should provide a detailed explanation of the proposed figures. For instance, Figures 14 and 15 represent the Discriminator Loss and Generator Loss, respectively. However, the current descriptions lack clarity and specificity. To make the figures more meaningful, the authors should descript relevant numerical values and offer a more comprehensive interpretation.

**Response:** Thank you for the observation. We have revised the manuscript to include a more detailed explanation of Figures 14 and 15 in section 4.3. Specifically, we now provide a clear description of the Discriminator Loss and Generator Loss trends, incorporating relevant numerical values to highlight key observations. Also, we have categorically scrutinized and provided a more comprehensive interpretation of all the figures.

**Reviewer #3: Comments**

The paper entitled “An authentication system based on MAC addresses for multilayered SDN intrusion detection using dual discriminator conditional GAN and growing self-organizing maps.” presents a novel four-Q curve authentication system based on MAC addresses for multilayered SDN intrusion detection system utilizing deep learning techniques to identify and prevent attacks. I have the following comments that could improve the paper's structure:

1. The abstract is satisfactory; however, it necessitates some enhancements. Authors should initially enhance the abstract's composition. Subsequently, it should explicitly illustrate the outcomes of the proposed scheme, including numerical values and percentages.

**Response:** Thank you for your insightful feedback. We have revised the abstract as per your suggestions. Additionally, the outcomes of the proposed scheme, including numerical values and percentages, have been added.

2. Authors should give additional consideration to certain aspects of the manuscript, as well as to the numerous errors and formatting issues that are present in the current version.

**Response:** Thank you for your suggestion. We have thoroughly checked the manuscript for the possible formatting issues and rectified the same as and when applicable.

3. The current manuscript contains several typos and formatting issues, along with several considerations the authors should take into account, such as: -Page 2: " Therefore, by implementing an effective IDS in MAC-based SDN system, adversaries can be reduced or eliminated". The authors should check for similar mistakes and revise them.

**Response:** Thank you for your suggestion. We have thoroughly checked the manuscript for the possible formatting and rectified the same as and when applicable.

4. I advise the authors to revise the quality of all figures.

**Response:** Thank you for your suggestion. We have improved the quality of all the figures and included in the revised manuscript (i.e., Figures files).

5. The proposed solution by the authors suggests the following: First, in the infrastructure layer, deep learning models get trained with more robustness. In addition, in the control plane layer, the local discriminator consists of a leaky ReLU activation function, two batch normalization layers, and five convolutional layers. The discriminator system includes four fully connected layers. Lastly, the application layer contains an unsupervised neural network of the GSOM type based on the SOM methodology. My question is, is it a practical solution to use all these complex techniques within each part? Please discuss in detail.

**Response:** Thank you for your feedback. We have considered your concern and acknowledge that the use of these complex techniques within each layer is practical which is provided in section 3.5.4. The proposed methods address specific challenges effectively, and their combination ensures robust performance across the system.

6. Authors are advised to compare their work with the literature in the result section.

**Response:** Thank you for your suggestion. We have included a comparative analysis of our work with relevant literature in the results section 4.3 (Table 8) to highlight the improvements and contributions of our proposed approach.

Additional Comments (From the Academic editor):

1. We note that Figure 1 in your submission contain copyrighted images. Figures must be replaced or redrawn.

**Response:** Thank you for the clarification. We understand that Figure 1 is an original figure created using Visio and does not contain any copyrighted content. However, we have included the new figure (i.e., Figure 1 in the Figures Files) instead of previous Figure 1.

2. Please include your full ethics statement in the ‘Methods’ section of your manuscript file. In your statement, please include the full name of the IRB or ethics committee who approved or waived your study, as well as whether or not you obtained informed written or verbal consent. If consent was waived for your study, please include this information in your statement as well.

**Response:** Thank you for your feedback. We will include the full ethics statement in the 'Methods' section of the manuscript. This will include the name of the IRB or ethics committee that approved or waived the study, as well as information regarding whether informed written or verbal consent was obtained, or if consent was waived for the study.

3. Please note that the Journal has specific guidelines on code sharing for submissions in which author-generated code underpins the findings in the manuscript. In these cases, all author-generated code must be made available without restrictions upon publication of the work.

**Response:** Thank you for your comment. We acknowledge the journal's guidelines regarding code sharing. We will ensure that all author-generated code underpinning the findings in our manuscript is made available without restrictions upon publication, in compliance with the journal's requirements.

4. Please ensure that your manuscript meets journal style requirements, including those for file naming.

**Response:** Thank you for your feedback. We will review and ensure that our manuscript fully adheres to the journal's style requirements, including the appropriate file naming conventions.
