# Supplementary material for: Multilayered SDN security with MAC authentication and GAN-based intrusion detection
Source: PLoS One. 2025 Sep 4;20(9):e0331470. doi: 10.1371/journal.pone.0331470 (PMC12410795; doi:10.1371/journal.pone.0331470)
Supplement: Attachment — Submitted filename: Reponse to Reviwer Comments 2 (Plos One).docx [file pone.0331470.s007.docx]

**Rebuttal Letter**

Dated: 05/02/2025

To

Academic Editor
PLOS ONE

Subject: Response to Reviewers' Comments for Manuscript titled: **“Multilayered SDN Security with MAC Authentication and GAN-Based Intrusion Detection**” with Manuscript Id: **PONE-D-24-24864R1**

Dear Dr. Muhammad Anwar,

We thank you and the reviewers for the opportunity to revise and resubmit our manuscript, titled “**Multilayered SDN Security with MAC Authentication and GAN-Based Intrusion Detection”** submitted to PLOS ONE. We appreciate the reviewers’ thoughtful and constructive comments, which have helped us improve the quality and clarity of our work. We have provided the detailed responses to each comment and explain the revisions made in the manuscript in the file titled ***Response to the Reviewer Comments*.**

We have addressed each comment in turn, and where necessary, we have highlighted the changes in the revised manuscript. Please find our point-by-point responses below. We have also made minor edits to improve clarity and coherence throughout the manuscript. These include rewording certain sentences and correcting typographical errors. We hope that our responses and the revised manuscript address the reviewers’ concerns satisfactorily. We are confident that the changes have significantly improved the paper and would make you give a decision in favour to our proposal. Thank you for your time and consideration, and we look forward to your feedback.

Sincerely,

Nanavath Kiran Singh Nayak, Dr. Budhaditya Bhattacharyya* (Corresponding Author)
School of Electronics Engineering,

Vellore Institute of Technology, Vellore, India.

[budhaditya@vit.ac.in](mailto:budhaditya@vit.ac.in)

+91-9955658860.

**Reviewer's Responses to Questions**

1. If the authors have adequately addressed your comments raised in a previous round of review and you feel that this manuscript is now acceptable for publication, you may indicate that here to bypass the “Comments to the Author” section, enter your conflict-of-interest statement in the “Confidential to Editor” section, and submit your "Accept" recommendation.

Reviewer #2: (No Response)

Reviewer #3: All comments have been addressed

**Response:** Thank you for your positive response.

2. Is the manuscript technically sound, and do the data support the conclusions?

The manuscript must describe a technically sound piece of scientific research with data that supports the conclusions. Experiments must have been conducted rigorously, with appropriate controls, replication, and sample sizes. The conclusions must be drawn appropriately based on the data presented.

Reviewer #2: Yes

Reviewer #3: Yes

**Response:** We sincerely appreciate your positive feedback.

3. Has the statistical analysis been performed appropriately and rigorously?

Reviewer #2: Yes

Reviewer #3: Yes

**Response:** Thank you for your positive feedback.

4. Have the authors made all data underlying the findings in their manuscript fully available?

The [PLOS Data policy](http://track.editorialmanager.com/CL0/http:%2F%2Fwww.plosone.org%2Fstatic%2Fpolicies.action%23sharing/1/010f01945e4005ed-12f7deab-99d6-4b12-a27b-f134c3536f4c-000000/qOpXdJpO2NJfcHDYJVxq93T-6gF6QBFTVDgWoYkvagU=193) requires authors to make all data underlying the findings described in their manuscript fully available without restriction, with rare exception (please refer to the Data Availability Statement in the manuscript PDF file). The data should be provided as part of the manuscript or its supporting information, or deposited to a public repository. For example, in addition to summary statistics, the data points behind means, medians and variance measures should be available. If there are restrictions on publicly sharing data—e.g. participant privacy or use of data from a third party—those must be specified.

Reviewer #2: No

Reviewer #3: Yes

**Response:** The authors appreciate the insightful observations made by the reviewers. All data underlying the findings in this manuscript have been made fully available accordance with the PLOS Data Policy. The data is provided as supplementary files and deposited in a public repository, e.g., GitHub, Kaggle. (https://github.com/abhinav-bhardwaj/Network-Intrusion-Detection-Using-Machine-Learning/blob/master/Data_Preprocessing_NSL_KDD.ipynb)] under the following link: [https://www.kaggle.com/code/abhaymudgal/intrusion-detection-system]. Also, the Zip file is attached related data.

5. Is the manuscript presented in an intelligible fashion and written in standard English?

PLOS ONE does not copyedit accepted manuscripts, so the language in submitted articles must be clear, correct, and unambiguous. Any typographical or grammatical errors should be corrected at revision, so please note any specific errors here.

Reviewer #2: No

Reviewer #3: Yes

Response: We have thoroughly checked the manuscript and all the necessary modification were made.

**6.** **Review Comments to the Author**

Please use the space provided to explain your answers to the questions above. You may also include additional comments for the author, including concerns about dual publication, research ethics, or publication ethics. (Please upload your review as an attachment if it exceeds 20,000 characters)

**Reviewer Comments #2:**

1. Why did the authors abbreviate Dual-Discriminator Conditional Generative Adversarial Network [36] as DC-GAN instead of DDcGAN?

**Response:** Thank you for your critical observation. The necessary changes have been made in the manuscript.

2. Authors should revise their paper carefully to avoid writing errors: e.g., single, and double inverted commas are used in a confusing way, why are there 'L_loss' and "L_loss"? The multiplication sign should not be replaced by word "x". In addition, the symbolic fonts of the equation need to be checked and revised again.

**Response:** Thank you for your insightful observation. We have made necessary changes in all the symbols/equations, wherever it is applicable.

3. Better to highlight the best performances in bold, or in a different colour (e.g. red) to make the tables easier to read.

**Response:** Thank you for your constructive feedback. We have highlighted our contribution in bold for all the tables.

Reviewer #3: The authors respond to every comment. Other than that, I have no concerns. The paper can be accepted as it is.

**Response:** Thank you for your positive feedback.
